# Supplementary material for: Formation and Maintenance of Robust Long-Term Information Storage in the Presence of Synaptic Turnover
Source: PLoS Comput Biol. 2015 Dec 29;11(12):e1004684. doi: 10.1371/journal.pcbi.1004684 (PMC4699846; doi:10.1371/journal.pcbi.1004684)
Supplement: S1 Text — Here we investigate, how the presented results generalize to a broader class of stationary distributions for learning and forgetting. (PDF) [file pcbi.1004684.s001.pdf]

Supporting Text S1
Formation and maintenance of robust long-term information storage in the presence of synaptic turnover

Fauth, Michael[1,2,*], Wörgötter, Florentin[1,2], and Tetzlaff, Christian[2,3,4]

[1]Third Physics Institute, Georg-August University, Göttingen, Germany
[2]Bernstein Center for Computational Neuroscience, Göttingen, Germany
[3]Max-Planck Institute for Dynamics and Self-Organization, Göttingen, Germany
[4]Department of Neurobiology, Weizmann Institute of Science, Rehovot, Israel
[*]mfauth@gwdg.de

Example distributions and deletion probabilities

for $\mu = 5$, $\sigma = 1.2$, $\lambda = 0.01$, $C = 0.1$ and $N = 10$ (Fig. 2, 3 and 5):

S	$p^{low}[S]$	$d^{low}[S]/b$	$p^{wp}[S]$	$d^{wp}[S]/b$	$p^{high}[S]$	$d^{high}[S]/b$
0	0,99005		8,91E-01		1,36E-08	
1	0,0099005	1,93E-02	8,91E-03	1,00E+03	7,03E-06	1,00E+03
2	4,95E-005	3,48E-02	1,35E-04	2,96E+02	9,08E-04	9,00E+02
3	1,65E-007	8,28E-02	2,92E-03	1,23E-01	2,92E-02	8,00E+02
4	4,13E-010	2,18E-01	2,35E-02	2,18E-01	2,35E-01	7,00E+02
5	8,25E-013	5,99E-01	4,70E-02	5,99E-01	4,70E-01	6,00E+02
6	1,38E-015	1,67E+00	2,35E-02	1,67E+00	2,35E-01	5,00E+02
7	1,96E-018	4,59E+00	2,92E-03	4,59E+00	2,92E-02	4,00E+02
8	2,46E-021	1,21E+01	9,08E-05	1,21E+01	9,08E-04	3,00E+02
9	2,73E-024	2,87E+01	7,03E-07	2,87E+01	7,03E-06	2,00E+02
10	2,73E-027	5,18E+01	1,36E-09	5,18E+01	1,36E-08	1,00E+02

Generalisation to other stationary distributions

The simulations shown in the main text are all conducted with three different stationary distributions, which constitute the three stimulation conditions. In the following, we will show that the results also hold for a broader class of stationary distributions.

Development of mutual information between $S(t)$ and $S(0)$ for other stationary distributions

First, we demonstrate that the long-term information storage is indeed a feature of the bimodal distribution and only depends on the probability at the minimum of the stationary distribution. For the *wp*-distribution we use in Figures 1-5, this minimum is at $S = 2$ (see table above). From this we can construct arbitrary stationary distributions with the same minimum, the same probability mass in the peaks ($1 - C$ and C), but very different shapes of the peak:

- A stationary distribution with $p[S = 2] = p^{wp}[S = 2]$ equal to the *wp*-distibution, but uniformly distributed below and above $S = 2$. This distribution has the broadest possible peaks while preserving the minimum at $S = 2$.
- A stationary distribution with all probabilities $p[S] = p^{wp}[S = 2]$ equal to the *wp*-distibution and the additional probability mass distributed to $S = 0$ and $S = 5$ with weights $1 - C$ and C. This distribution has very sharp peaks.

Furthermore, we added two other special cases, which do not have a minimum:

- the distribution emerging from $d[S] = b = const$ and
- a uniform stationary distribution.

For each of those distributions, we simulated the development of mutual information between the number of synapses at time t and the initial conditions for a population of connections. It can be seen in Figure S1 that for the latter two distributions, mutual information decays after 10^7 to 10^8 time steps. This is comparable to the results from the *high* and *low* distributions and indicate that no long-term information storage takes place.

On the other hand, all other distributions with a minimum at $p[S = 2] = p^{wp}[S = 2]$ exhibit long-term information storage on a comparable time scale of $10^{10} - 10^{11}$ time steps. This shows that the shape of the peaks does not influence the long-term information storage as long as the minimum of the stationary distribution has the same probability. Note, by construction, the distribution with the sharp peaks has multiple states with the same minimal probability, which even prolongs the storage time.

Learning with other stationary distributions

A similar effect can be observed, when the learning experiment from Figure 5 is repeated with altered stationary distributions during the learning phase. We again used the probability minimum of the *wp*-distribution at $S = 2$. From this we constructed the following distributions:

- A High distribution with a broad peak (Fig. S2A): $p[S] = p^{wp}[S = 2]$ for $S = 0..2$ and the rest of the probability mass uniformly distributed among $S > 2$.
- A Low distribution with a broad peak (Fig. S2A): $p[S] = p^{wp}[S = 2]$ for $S = 2...N$ and the rest of the probability mass uniformly distributed among $S < 2$.

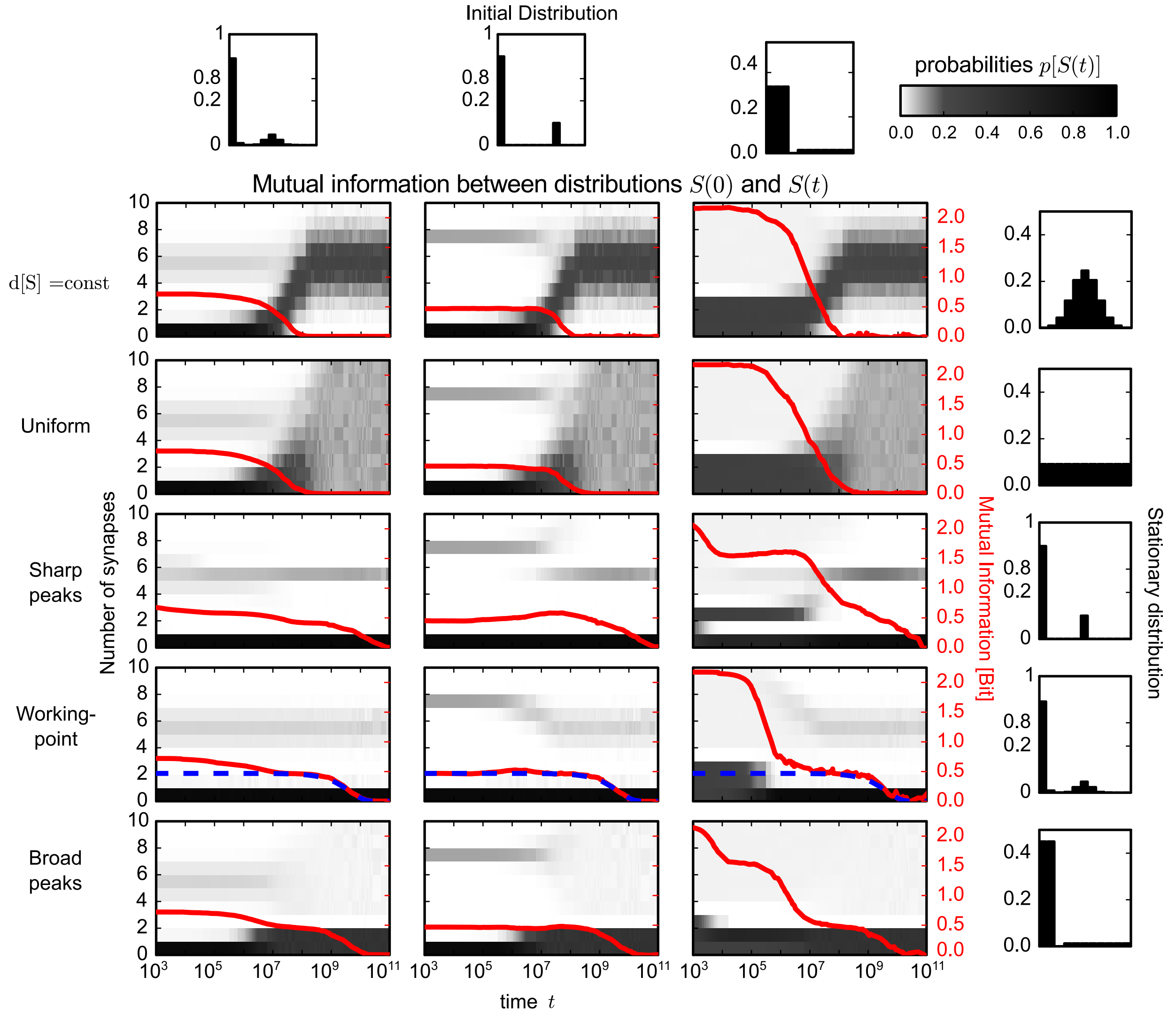

Figure S1: **Development of mutual information under special stationary distributions given different initial conditions.** Same as Figure 3 but for the following stationary distributions: (1^{rst} row) distribution resulting from $d[S] = b$; (2nd row) uniform distribution; (3rd row) distribution with the same minimum as the *wp*-distribution, but much sharper peaks; (4th row) the *wp*-distribution; (5th row) distribution with the same minimum as the *wp*-distribution, but much broader peaks. All bimodal distributions with the same minimum exhibit comparable long-term information storage, while the mutual information for the other distributions decays on the same time scale as for *high* or *low* stimulation conditions.

- A High distribution with a very narrow peak (Fig. S2C): $p[S] = p^{wp}[S = 2]$ for $S \neq 5$ and the rest of the probability mass accumulated at $S = 5$.
- A Low distribution with a very narrow peak (Fig. S2C): $p[S] = p^{wp}[S = 2]$ for $S \neq 0$ and the rest of the probability mass accumulated at $S = 0$.
- A High distribution with a very narrow peak (Fig. S2D): $p[S] = p^{wp}[S = 2]$ for $S \neq 3$ and the rest of the probability mass accumulated at $S = 3$.
- A Low distribution with a very narrow peak (Fig. S2D): $p[S] = p^{wp}[S = 2]$ for $S \neq 1$ and the rest of the probability mass accumulated at $S = 1$.

In Figure S2 we compare the accumulation and decay of mutual information between the number of synapses and the applied stimulation condition for stimuli with narrow (Fig. S2C and D) or broad peaks (Fig. S2A) with the distributions used in the main text (Fig. S2B). For each set of *high* and *low* distributions, connections receive *high* or *low* stimulation with probability of 50% .

It can be seen that these altered distribution shapes lead to a decreased learning speed of about one order of magnitude compared to the *high* and *low* distributions from the main text. This can intuitively be explained by the fact that for the uniform parts of the altered *high* and *low* distributions, synapse creation and deletion are approximately equally probable. Therefore, the connection in principle undergoes an unbiased random walk. For the *high* and *low* distributions used in the main text, there is no uniform region and either building or deletion are more probable. This effectively introduces a drift in the random walk. During a *high* (*low*) stimulation, connections which are in the lower (upper) peak have to make a transition to the upper (lower) peak. As the *high* (*low*) distribution has decreasing probabilities towards the lower (upper) peak, it introduces a drift towards the upper (lower) peak, which leads to a faster transition to the other peak and, thus, to a faster convergence to the stationary distribution. Also the width of the uniform region of the distribution contributes to this phenomenon. It can be seen from Figure S2 that moving the sharp peaks to the edges of the broad peaks yields approximately the same timescale for learning (Fig. S2A and D), but moving the sharp peaks away from the edges slows learning down (Fig. S2C)

However, the time scale for information retention under *wp*-conditions is still longer (see also Fig. S1). This happens due to the fact that the stationary distributions also introduces a drift, which drives the connections away from the minimum and makes peak transitions less probable. This intuitive explanation and the simulation results demonstrate that the presented results should qualitatively hold for much broader class of stationary distributions than used in the main text.

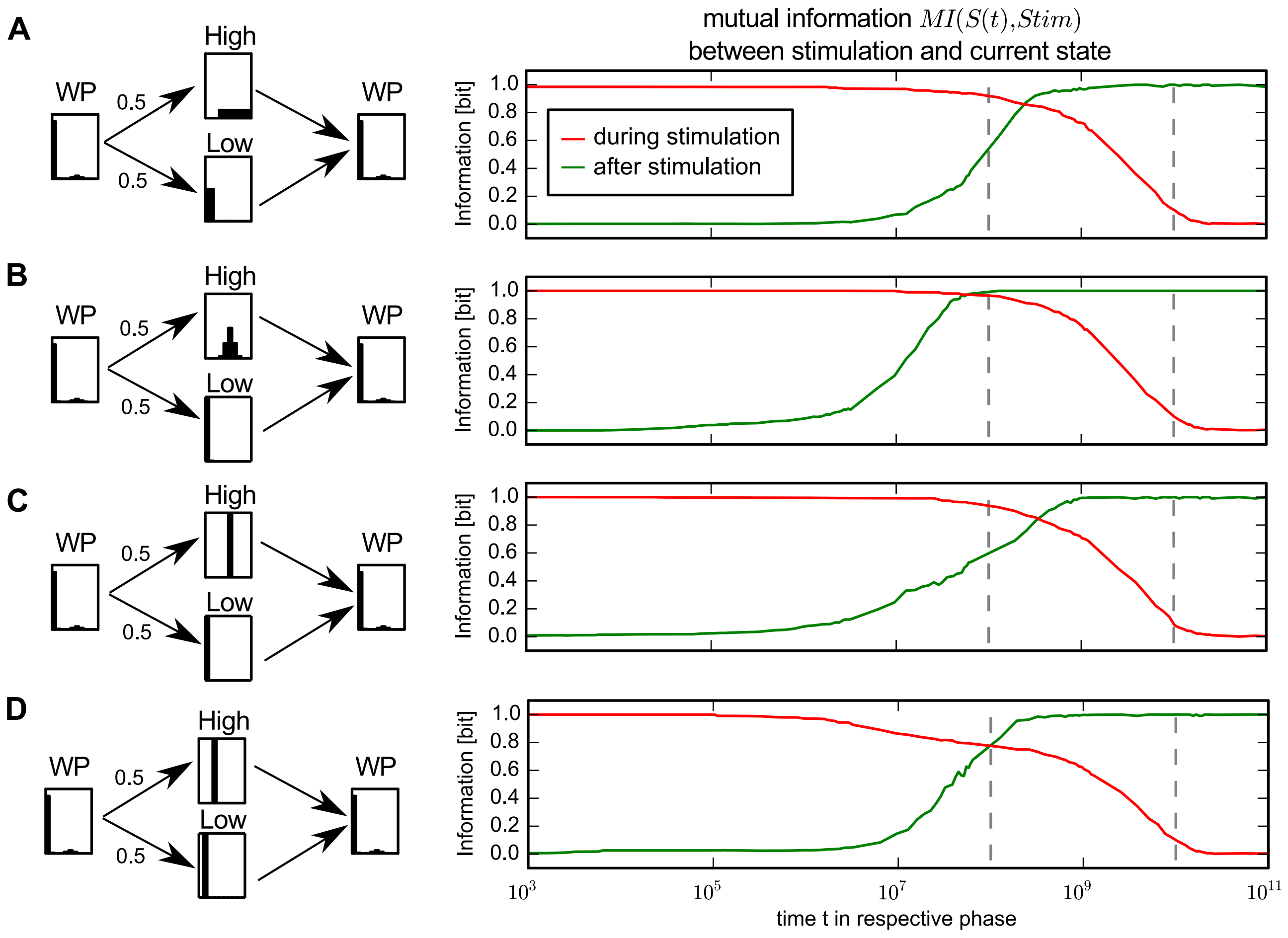

Figure S2: **Changed shape of the stationary distribution during learning alters learning speed, but still yields faster learning than forgetting.** Same as Figure 5A and C, but for *high* and *low* distribution with different peak shapes: (A) broadest possible peaks (B)for comparison, peak shape as used in main text (C) most narrow peaks at $S = 0$ and 5, (D) most narrow peaks at $S = 1$ and 3

Discernibility of similar stimulation conditions

In the following we investigate how the proposed mechanism for long-term storage of information influences how stationary distributions, which are used for learning, can be distinguished on long time scales when the connections are set back to *wp*-condition.

As we show in Figure 3, the information, which can be preserved on long time scales, corresponds to the peak in which a connection was at the beginning of the measurement. As the *wp*-distribution has only two peaks, it can be expected that, on the long run, it is possible to distinguish the influence of maximally two different stationary distributions applied in the learning phase. These distributions must have different overlap with the two peaks of the *wp*-distribution (i.e. different numbers of connections which are in the upper / lower peak regime after learning) such that, after learning, the probability to start in the upper peak (or lower peak respectively) differs. The *high* and *low* distributions used in the main article can be considered as the extreme cases which only overlap with one of the peaks. A third distribution would, in terms of probability mass in each peak, not be linearly independent from the others. Thus, learning two different stimulation conditions leads to the best possible discrimination between them.

Therefore, we simplify the experiment from Figure 5. All connections are still in *wp*-conditions in the first and third phase (retention phase) of the experiment. However, in the second phase (learning phase), we tune the connections to one of two stationary distributions with a 50%-chance.

From simulations of a population of connections, we can assess two important quantities:

- The mutual information between stimulus and number of synapses directly after learning provides a measure how well one can distinguish which stimulation condition a connection received. The distribution of the two stimulation conditions has an entropy of 1bit. This also constitutes the maximal possible mutual information between stimulus and number of synapses. When mutual information becomes smaller than 1bit, a single synapse would not provide sufficient information to decide which stimulation was present. In this case it is plausible that the information about a stimulation is reconstructed from a population of synapses.

  Note, when the connections reached their stationary distribution, this quantity does not depend on the *wp*-distribution, but only on the distributions used for learning.

- The time course of mutual information, on the other hand, shows how long the two stimulation conditions can be distinguished. As an estimate of the time scale we use the time T_{50} after which the mutual information has decreased to half of its initial value during the retention phase.

With these measures we will demonstrate that different probability masses in each peak (of the *wp*-distribution) are indeed the major criterion for the long-term discernibility of two distributions.

Two unimodal distributions: In a first simulation, we apply the *high*-stimulation used in the main text ($\mu = 5, \sigma = 1.2, C = 1$) and another *high*-stimulation with a different μ during the learning phase. Figure S3 shows that the more similar the two distributions become ($\Delta\mu \to 0$), the less mutual information is acquired after learning. Around $\Delta\mu = 0$, the mutual information approaches zero and is too low to determine T_{50} (discontinuity of the black curve). The further apart the peaks of the two distributions are, the more mutual information is observed after learning. In this case, it almost reaches the maximum possible value for $|\Delta\mu| \to 5$.

However, the time scale until this mutual information decays is highly different and strongly influenced by the shape of the *working-point* distribution. In general, when both distributions have a strong overlap with the upper peak of the *wp*-distribution ($\Delta\mu > -2$), the mutual information decays quickly. Long-term information storage occurs only for very low (negative) $\Delta\mu < -2$, when the the altered *high*-distribution has a strong overlap with the lower peak of the *wp*-distribution and becomes more similar to the *low*-stimulation condition.

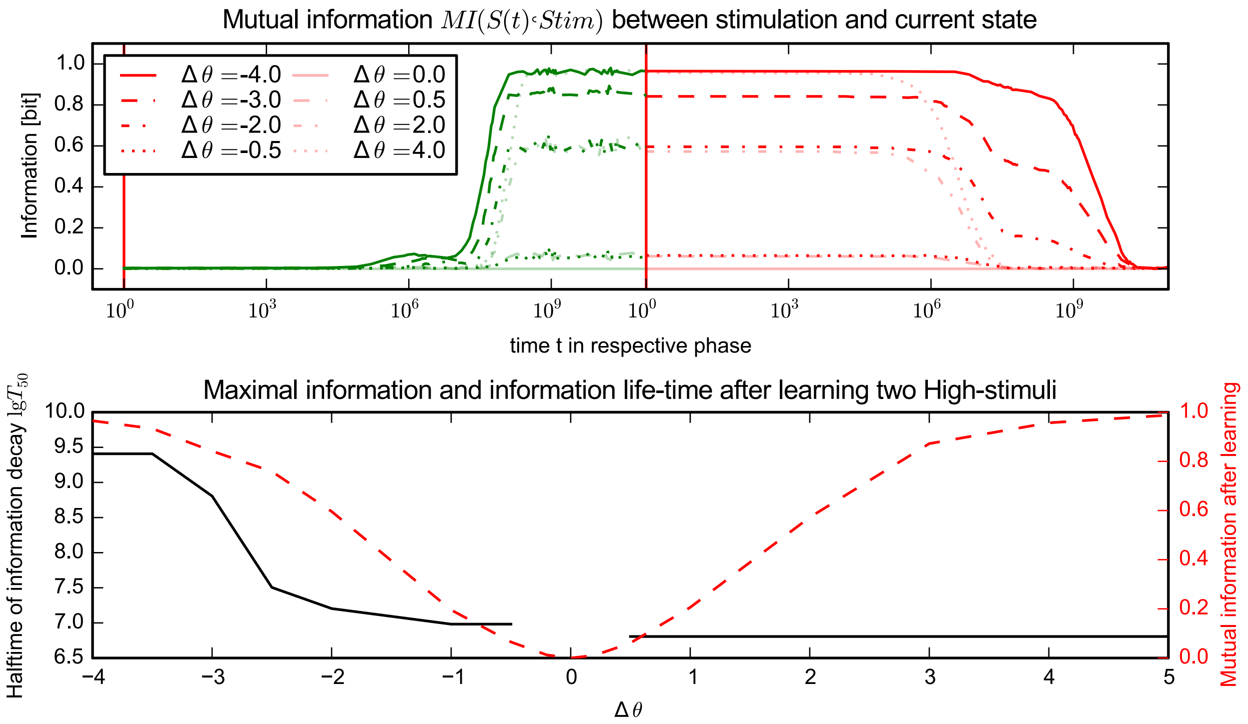

Figure S3: **Stimulation conditions with similar numbers of connections in each peak can only be distinguished for short times.** (Upper panel) The temporal development of the mutual information between stimulation condition and the number of synapses during (green) and after (red, wp-condition) presentation of two $High$-type stimulation conditions with peaks locations μ and $\mu + \Delta\mu$. (Lower panel) The time until the mutual information has decayed to 50% of its initial value after learning T_{50} (black) and the value of mutual information after learning (red).
Long-term information storage ($T_{50} > 10^8$) is only possible, when the alternative $High$ distribution has a significant overlap with the lower peak of the wp distribution. Note, around $\Delta\mu = 0$ the value after learning is too low to evaluate T_{50}.

The fact, that two distributions which only overlap with the upper peak of the wp-distributions can not be distinguished for long time scales, illustrates that only the overlap with the peaks of the wp-distribution influences the long-term discernibility of stimuli.

As this overlap is regulated by the parameter C, we expect that stimuli with distributions with different C-values should always be distinguishable for long times. This hypothesis will be tested in the following.

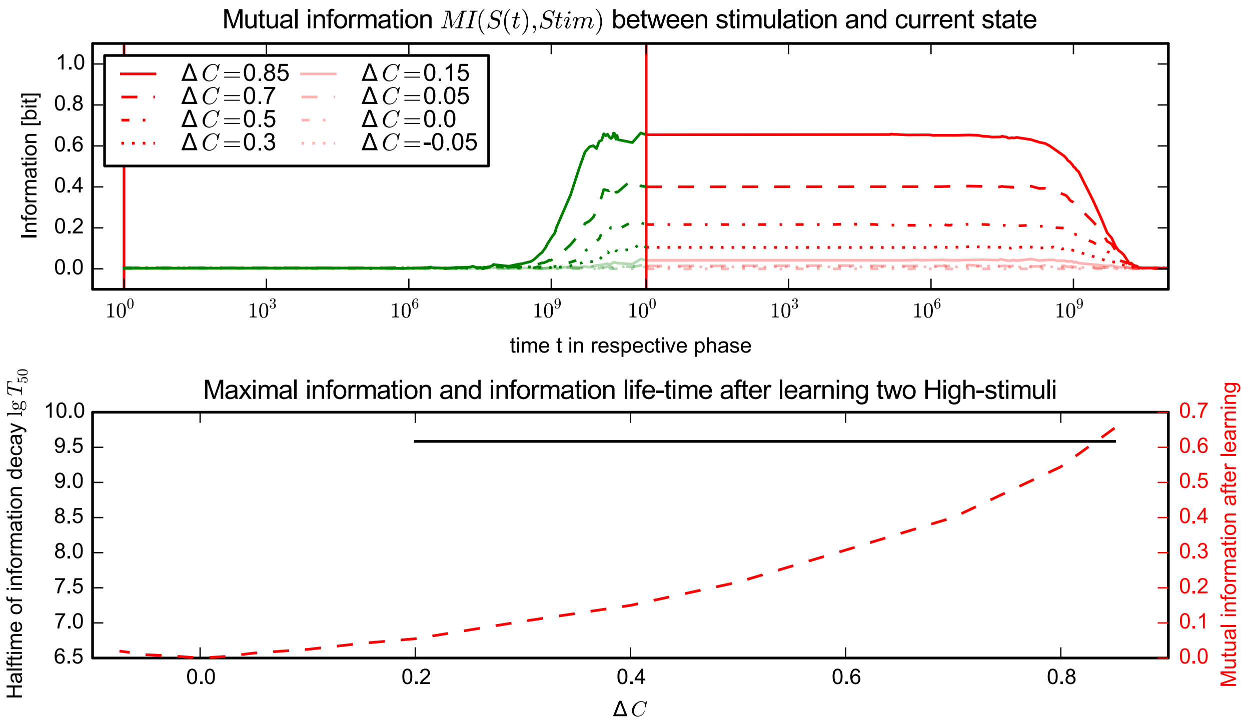

Figure S4: **Stimulation conditions with different numbers of connections in each peak can be distinguished for long times.** Same as Figure S3, but for the presentation of two *wp*-type stimulation conditions with weighting parameters C and $C + \Delta C$.
Around $\Delta C = 0$ the value after learning is also here too low to evaluate T_{50}.

Two bimodal distributions: In this experiment, we apply two *Working-point* distributions during the learning phase. One of them is the *wp*-distribution used in Figures 1-5 ($\mu = 5, \sigma = 1.2, C = 0.1$) and the other one uses a varied C.

Again, the acquired mutual information at the beginning of the learning phase is close to zero for small changes ΔC, i.e. for very similar distributions (Fig. S4). The larger the change in C the larger the mutual information, although the maximal mutual information of 1bit is not reached. This can be intuitively explained by the fact that one cannot distinguish, e.g., connections in the upper peak of the original *wp*-distribution from those from the upper peak of the altered *wp*-distribution (and this holds for at least 10% of the connections as $C = 0.1$).

However, importantly, it can be seen that the mutual information which is acquired persists for very long time scales (Fig. S4). Taken together, this means that connections could sense deviations in the balance between lower and upper peak and store them for very long times. To decide which stimulation condition was active one would however need a population of connections.
