# Supplementary material for: Formation and Maintenance of Robust Long-Term Information Storage in the Presence of Synaptic Turnover
Source: PLoS Comput Biol. 2015 Dec 29;11(12):e1004684. doi: 10.1371/journal.pcbi.1004684 (PMC4699846; doi:10.1371/journal.pcbi.1004684)
Supplement: S2 Text — (PDF) [file pcbi.1004684.s002.pdf]

Supporting Text S2
Formation and maintenance of robust long-term information storage in the presence of synaptic turnover

Fauth, Michael[1,2,*], Wörgötter, Florentin[1,2], and Tetzlaff, Christian[2,3,4]

[1]Third Physics Institute, Georg-August University, Göttingen, Germany
[2]Bernstein Center for Computational Neuroscience, Göttingen, Germany
[3]Max-Planck Institute for Dynamics and Self-Organization, Göttingen, Germany
[4]Department of Neurobiology, Weizmann Institute of Science, Rehovot, Israel
[*]mfauth@gwdg.de

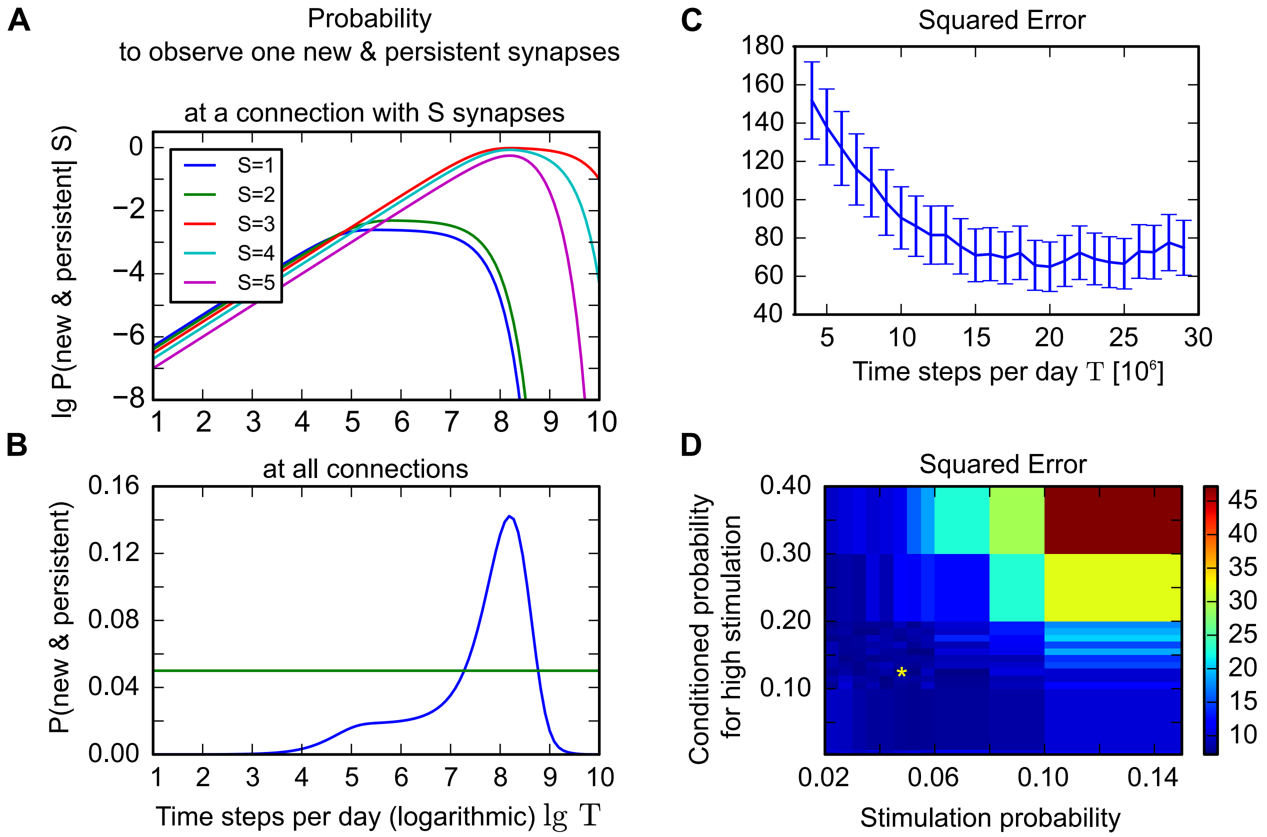

Figure S5: **Analytical predictions and simulations of number of best matching number of time steps per day** T (A) Probability that a synapse is formed within and persists until the end of a time interval $[0, T]$ at a connection which has S connections after formation. (B) Expected relative proportion of new synapses, i.e., the expected value that synapses are formed and persist until the end of a time interval $[0, T]$, divided by the expected number of preexisting synapses. The constant green line corresponds to the experimental value of 5%. The (first) intersection of both curves at $23 \cdot 10^6$ is the theoretical prediction for T (C) Squared error between model and the experimental data of early phase synapse creation in control group. The minimum of the error is consistent with the theoretically predicted value of $T = 23 \cdot 10^6$ time steps. Error bars are standard deviations of the error from 18 repeated simulations. (D) Squared error for the spine creation at day 16 of the early training phase (retraining paradigm) and the late phase spine turnover (all paradigms). Small errors are achieved, when the number of stimulated neurons and the proportion of neurons with *high* stimulation are low (under 10%). The best matching set of stimulation probabilities (lowest error) is marked by a yellow star.
